# Actinomyces neuii Causing Right-Sided Infective Endocarditis: A Case-Based Literature Review

**DOI:** 10.7759/cureus.63762

**Published:** 2024-07-03

**Authors:** Muhammad A Zaman, Sidra Kalsoom, Latasha Naidu

**Affiliations:** 1 Internal Medicine, Conemaugh Memorial Medical Center, Johnstown, USA; 2 Infectious Disease, Conemaugh Memorial Medical Center, Johnstown, USA

**Keywords:** right-sided infective endocarditis, native valve, infective endocarditis, winkia neuii, actinomyces neuii

## Abstract

*Actinomyces neuii* (also known as *Winkia neuii* nowadays), quite different from its genus,is a facultatively anaerobic organism that rarely causes human infections.Like the rest of its genus, it usually has a good prognosis. In this case report, we present an interesting case of a middle-aged female who presented to the emergency department (ED) with fever and dyspnea, eventually diagnosed with infective endocarditis (IE) caused by *A. neuii.* To the best of our knowledge, it is the first reported case of *A. neuii* causing right-sided infective endocarditis in a middle-aged female with no residual or prosthetic valvular disease.

## Introduction

Gram-positive bacilli, specifically *A. neuii*, were once regarded as contaminants but are now emerging as unusual organisms causing infections, including infective endocarditis (IE). *Actinomyces* species, characterized by their indolent nature, can cause a spectrum of granulomatous infections. These infections can involve the endovascular system or manifest in the orocervicofacial, thoracic, abdominopelvic, or central nervous system regions [1]. This extended incubation period underscores the critical time-sensitive nature of obtaining blood cultures. Fortunately, advancements in diagnostic techniques have facilitated the identification of *Actinomyces*. Enzyme-linked immunosorbent assays (ELISAs), indirect hemagglutination assays, and matrix-assisted laser desorption ionization time-of-flight mass spectrometry (MALDI-TOF MS) now offer more efficient diagnostic tools [2].

However, it is crucial to remember that *Actinomyces* is a slow-growing organism, often requiring 2-3 weeks for culture confirmation. *Actinomyces* infections require prolonged antibiotic therapy due to the slow-growing nature of the bacteria and their ability to form deep-seated abscesses and tissue involvement. The recommended duration of treatment for *Actinomyces* infections is typically several weeks to even months. In the case of infective endocarditis, the treatment is usually six weeks; however, the exact duration of treatment depends on multiple factors: whether the patient underwent any surgical management of infective endocarditis or not; recent and, if any, prior microbiological culture and sensitivity analyses; patient-specific risk factors; and specific geographic locations impacting the antibiogram.

## Case presentation

Initial presentation

A middle-aged female visited the hemodialysis center for her scheduled outpatient hemodialysis. During the pre-hemodialysis physical examination, she was noted to have a fever and minimal dyspnea. She was sent to the emergency department (ED) from the hemodialysis center. On arrival at the ED, the patient endorsed subjective fever and not feeling well for the last two days. The pertinent medical history included end-stage renal disease requiring weekly hemodialysis, hypertension, type 2 diabetes mellitus, schizoaffective disorder, severe peripheral artery disease, and fibromyalgia. She also had a right internal jugular tunneled hemodialysis catheter (Rt IJ-HDC) for hemodialysis. The initial vital signs were a temperature of 38.3°C (101°F), heart rate of 98 beats per minute, blood pressure of 136/86 mmHg, and respiratory rate of 20 breaths per minute.

Diagnostic workup

Physical examination was unremarkable, with no apparent skin changes at the Rt IJ-HDC insertion site or the left upper extremity brachiocephalic arteriovenous fistula (LUE BC AVF). The currently used Rt IJ-HDC had been replaced one month before presentation. The LUE BC AVF, created two years prior, was never used due to poor blood flow. The patient reported no other complaints. Initial day 1 laboratory workup revealed leukocytosis of white cell count 12.3 × 10^3^/uL and neutrophil-to-lymphocyte ratio of 18.9% (neutrophil: 85.3%, lymphocytes: 4.5%). Table 1 summarizes the key complete blood count results throughout index hospitalization. The dialysis center obtained blood cultures prior to transferring the patient to the ED. Considering the patient's immunosuppressed state, positive systemic inflammatory response syndrome, and concerns for sepsis of unknown origin, he underwent imaging studies, including computed tomography of the chest, abdomen, and pelvis, along with a transthoracic echocardiogram (TTE). All imaging studies were unremarkable, excluding any acute pathology.

**Table 1:** Key complete blood count results throughout index hospitalization RDW: red cell distribution width

	Reference	Day 1	Day 5	Day 10	Day 15
White blood cell count	3.10-8.50 × 10^3^/uL	12.33 × 10^3^/uL	8.81 × 10^3^/uL	11.9 × 10^3^/uL	8.34 × 10^3^/uL
Red blood cell count	4.50-6.30 × 10^6^/uL	2.92 × 10^6^/uL	2.49 × 10^6^/uL	2.56 × 10^6^/uL	2.13 × 10^6^/uL
Hemoglobin	14-18 g/dL	9.9 g/dL	8.3 g/dL	8.5 g/dL	7 g/dL
Hematocrit	40%-54%	31%	27%	28%	23%
Mean corpuscular volume	82-101 fL	105 fL	108 fL	109 fL	107 fL
Platelets	140-440 × 10^3^/uL	107 × 10^3^/uL	135 × 10^3^/uL	169 × 10^3^/uL	137 × 10^3^/uL
RDW	11.5%-14.5%	14.41%	14.5%	14.6%	14.6%
Neutrophils	38%-70%	85.3%	-	-	70.2%
Lymphocytes	0.9-2.9 × 10^3^/uL	4.5 × 10^3^/uL	-	-	18.1 × 10^3^/uL

Antibiotic management

Based on the clinical presentation and suspicion of sepsis, broad-spectrum intravenous antibiotic therapy was initiated with vancomycin (1.25 g daily) and piperacillin-tazobactam (4.5 g every six hours). Vancomycin dosing was adjusted to achieve target trough levels of 15-20 μg/mL. However, upon negative blood cultures within 48 hours of admission and following consultation with the infectious disease team, antibiotics were discontinued despite persistent fever. On the fourth day of hospitalization, gram staining of blood cultures obtained at both the dialysis center and the hospital (collected 12 hours apart) revealed gram-positive rods. Subsequent blood cultures from both facilities demonstrated growth of* A. neuii *in two out of four bottles (aerobic and anaerobic) on day 5. Notably, blood cultures were drawn using an aseptic technique from separate peripheral sites at both locations, with each sample consisting of four bottles (two aerobic and two anaerobic) sent for microbiological analysis. The in vitro susceptibility testing indicated pan-sensitivity of the cultured *A. neuii *isolate; however, the patient reported severe allergies to penicillin, cephalosporins, and sulfa drugs. Consequently, ertapenem therapy was started based on a multidisciplinary team clinical decision, at a dosage of 500 mg intravenously every 24 hours. The tunneled Rt IJ-HDC was removed to mitigate the possible source of infection, and the tip of the catheter was sent for further microbiological analysis. The Rt IJ-HDC removal and subsequent culture of its tip was a clinical decision, as the patient was septic in appearance and the Rt IJ-HDC was the only foreign body implant the patient had at that time. A new temporary catheter was placed in the left internal jugular tunneled hemodialysis catheter (Lt IJ-HDC) to facilitate ongoing hemodialysis sessions.

Surgical and interventional procedures

**Figure 1:**
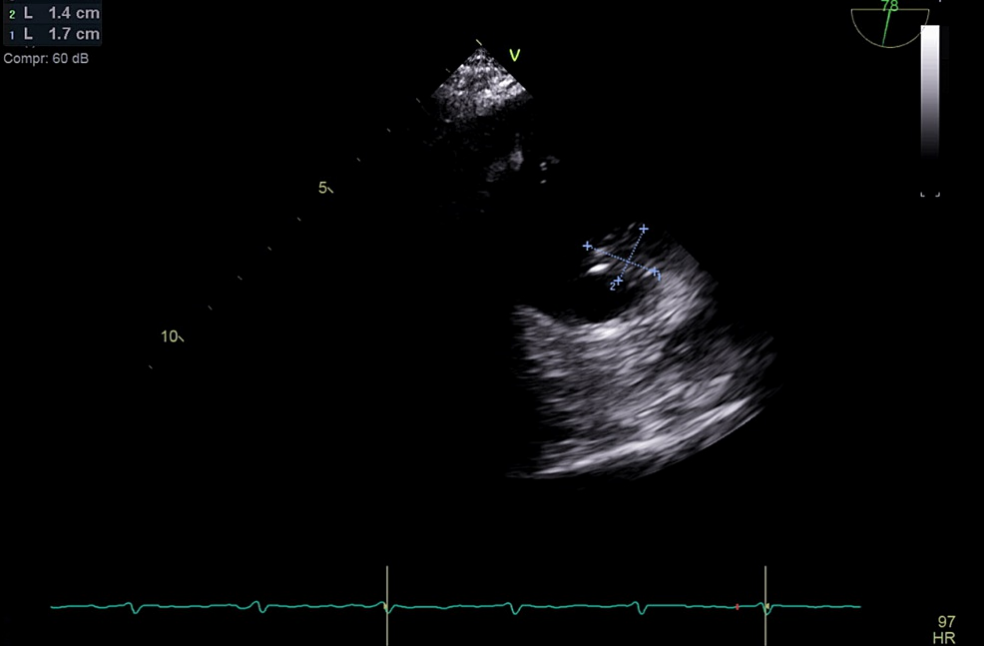
Transesophageal echocardiography demonstrates a vegetation measuring 1.4 × 1.7 cm in the right atrium

**Figure 2:**
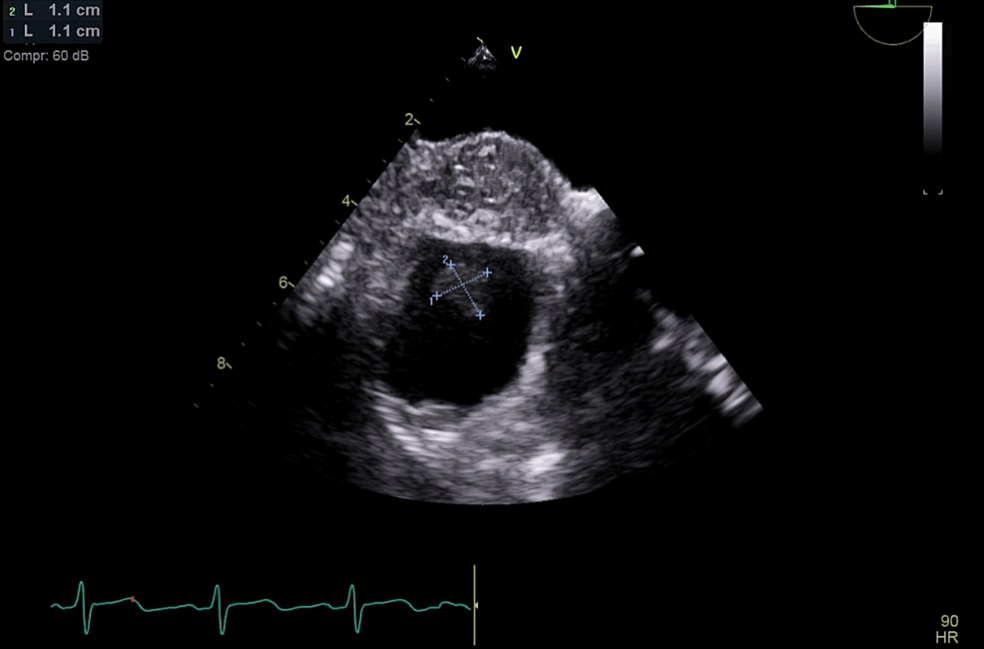
Repeat transesophageal echocardiography after eight weeks revealed a decreasing size of the vegetation

The repeated positive blood culture prompted physicians to do transesophageal echocardiography (TEE) despite negative TTE as the suspicion of infective endocarditis was high on differential diagnoses. The TEE revealed significant vegetation measuring 1.4 × 1.7 cm at the junction of the right atrium and superior vena cava (Figure 1). Based on these findings suggestive of *A. neuii *infective endocarditis, the ertapenem dosage was increased from 500 mg to 1 g intravenously daily based on culture results and clinical response. Additionally, the temporary Lt IJ-HDC, which was found to be positioned near the vegetation, was also removed to minimize the risk of further thrombogenesis. The left upper extremity brachiocephalic arteriovenous fistula (LUE BC AVF) was successfully cannulated on two separate occasions for hemodialysis. Five days after the removal of the temporary Lt IJ-HDC, the vascular team successfully performed a transposition of the LUE BC AVF with the aid of a left upper extremity fistulogram and balloon angioplasty of the cephalic vein stenosis. The patient received the scheduled hemodialysis sessions through LUE BC ACF throughout hospitalization. A subsequent TEE conducted eight weeks later demonstrated a reduction in vegetation size (Figure 2). The patient's recovery progressed without complications. While no further TEE was performed per the patient's preference, a TTE performed three months, after the second TEE, revealed no concerns for any residual vegetations, hence not requiring any further TEE.

Follow-up and long-term outcome

The patient completed six weeks of 1 g IV ertapenem. The patient's laboratory workup on the day of discharge (day 15) revealed a white blood cell count of 8.34 × 10^3^/uL and neutrophil-to-lymphocyte ratio of 3.8% (neutrophil: 70.2%, lymphocytes: 18.1%). Of note, the patient's hemoglobin was 7 mg/dL at the time of discharge, which was attributed to anemia of chronic disease compounded by iatrogenic anemia. The patient was readmitted after two months of hospital discharge due to LUE BC AVF malfunction (compromised blood flow). This necessitated the placement of a temporary right femoral HDC, later followed by the implantation of a new tunneled Rt IJ-HDC. We did not notice any specific role in the management or outcome that was influenced by fibromyalgia; however, multiple vascular surgery procedures and AVF malfunctions can be attributed to the patient's medical history of severe peripheral arterial disease. No long-term antibiotic therapy or other management strategies post-discharge to prevent recurrence was started, and the patient was followed up for two years overall with no complications or recurrence noted.

## Discussion

Microbiology

*Actinomyces* species are typically anaerobic, gram-positive, branching filamentous rods. However, *A. neuii *deviates from its genus by exhibiting non-granulomatous, non-branching morphology and displaying aerobic or aerotolerant growth characteristics [3,4]. Notably, *A. neuii *has recently been reclassified as *Winkia neuii*, named to honor Harold Neu, an authority in antimicrobial chemotherapy and infectious diseases [5]. However, both names are currently used interchangeably. Unlike classical actinomycosis, infections caused by *A. neuii* typically do not present with abscess formation or the characteristic discharge of sulfur granules from sinuses. Due to its extremely slow growth rate, cultures for *A. neuii* require extended incubation periods of 2-3 weeks for accurate detection. Humans serve as the sole natural reservoir for *Actinomyces* species, which are not prevalent in the environment. These organisms typically exploit tissue injuries or mucosal breaches for invasion and rarely disseminate via the bloodstream. If a clinician suspects actinomycosis, consulting with an infectious disease specialist is highly recommended [6,7].

*Actinomyces neuii* and infective endocarditis

To the best of our knowledge, it is the first reported case of *A. neuii *causing right-sided infective endocarditis in a patient with no residual or prosthetic valvular disease or intravenous drug use. In this case, we encountered two significant challenges. The first challenge was the rarity of the organism causing right-sided endocarditis, making it a rare bug's disease at a rare location. The second challenge was the patient's history of severe allergic reactions, making it more challenging to select optimal medical therapy. Primary IE caused by *Actinomyces* is a rare occurrence, with approximately 100 documented cases reported in the literature [8-10]. However, the past decade has witnessed a surge in case reports detailing *Actinomyces* species causing left-sided IE, particularly in patients with specific risk factors such as recent dental procedures, prosthetic valve endocarditis, or a history of intravenous drug abuse [11]. Notably, all reported cases of right-sided IE involving the tricuspid valve have been strongly associated with intravenous drug use [12]. The clinical course of *Actinomyces*-induced IE is characterized by indolence and appears to remain relatively stable over time [13]. Fortunately, advancements in treatment strategies have demonstrated improved outcomes, reducing mortality and complication rates associated with this infection. It is noteworthy that the classic physical examination findings associated with acute and subacute endocarditis, such as Janeway lesions, splinter hemorrhages, Osler nodes, and rose spots, are typically absent in patients with *Actinomyces* IE. Cardiac auscultation also often yields unremarkable findings [14]. While *A. neuii *has been implicated in only two documented cases of infective endocarditis (IE), both instances involved pre-existing valvular heart disease (one with a bicuspid aortic valve and the other with a prosthetic aortic valve) [15]. Notably, 12 additional cases of *A. neuii *bacteremia have been reported, excluding those associated with IE. It remains unclear whether bacteremia arising from frequent endovascular cannulations in these patients subsequently caused IE or, conversely, if the IE originating from HDC access resulted in the bacteremia [15].

A new technology based on clinical proteomics, MALDI-TOF MS, is one of the established diagnostic modalities that offer accurate and rapid methods of identifying and diagnosing actinomycosis; however, it cannot differentiate between different types of *Actinomyces*. The other newer promising molecular techniques such as nucleic acid probes and polymerase chain reactions can diagnose *Actinomyces* more rapidly than culture results and have the potential to be more specific and sensitive than cultures respectively; however, they are still under development. Considering the slow growth associated with *Actinomyces*, these rapid diagnostic modalities that would enable us to diagnose masses rapidly would be a game changer.

Treatment and prognosis

Due to the rarity of cases, there are no guidelines about optimal antibiotic choice and duration of therapy, as well as alternative options in case of drug allergy/intolerance of antibiotics. As of now, the treatment regimen and choice of antibiotics for *Actinomyces*-induced IE mirror those employed for non-cardiac actinomycosis infections. In terms of susceptibility, *A. neuii *demonstrates a similar coverage spectrum as other *Actinomyces* species. Penicillin or beta-lactam antibiotics serve as the first-line treatment. For patients with penicillin allergies, alternative options such as erythromycin, clindamycin, tetracyclines, or vancomycin may be considered. Gentamicin and ciprofloxacin exhibit minimal efficacy against *A. neuii.* It is important to note that while susceptible to antibiotics, these organisms demonstrate a poor response to metronidazole [16-18]. Pre-existing valvular damage from prior IE or a compromised immune system is hypothesized to contribute to a more aggressive course of infection by the pathogen. This may have been the case with our patients as well. Earlier studies suggested that *Actinomyces* infections are likely endogenous, with the organism exhibiting an affinity for atheroma. Delayed diagnosis is a frequent occurrence in *Actinomyces* endocarditis and other infections caused by this genus. However, with advancements in laboratory techniques and a growing body of case reports, clinicians should exercise heightened vigilance regarding infections associated with *Actinomyces*, particularly those involving endovascular involvement [19,20].

## Conclusions

While *Actinomyces* infections typically exhibit an indolent course, the growing prevalence of these infections necessitates heightened awareness among physicians considering its atypical presentation.

In our case, blood cultures taken at the dialysis center proved very beneficial. A timely diagnosis can lead to reduced hospital stays, improved patient satisfaction, and potentially more efficient treatment due to faster diagnosis. The slow growth of *Actinomyces* poses a diagnostic challenge that can be overcome by newer promising under-investigation molecular techniques such as nucleic acid probes and polymerase chain reactions. Due to its rarity and no guidelines about optimal treatment, duration, and choice of antibiotics, a proactive approach that fosters collaboration between various healthcare disciplines, particularly with infectious disease specialists, is crucial for optimal patient outcomes in managing such cases.
